# Supplementary material for: Chemical Mechanisms Underlying Sweetness Enhancement During Processing of Rehmanniae Radix: Carbohydrate Hydrolysis, Degradation of Bitter Compounds, and Interaction with Taste Receptors
Source: Foods. 2025 Nov 17;14(22):3932. doi: 10.3390/foods14223932 (PMC12651911; doi:10.3390/foods14223932)
Supplement: Supplementary file 1 [file foods-14-03932-s001.zip › foods-3911440-supplementary.pdf]

Table S1. Absolute sugar contents in different processed products of Rehmanniae Radix.

Batches	Fru mg/g	Glu mg/g	Suc mg/g	Mel mg/g	Raf mg/g	Mnt mg/g	Sta mg/g
R1	41.00	37.00	118.25	20.50	88.00	87.50	424.75
R2	33.75	37.25	122.00	24.00	98.50	73.25	409.50
R3	15.75	21.25	156.25	10.00	89.50	32.25	443.20
R4	14.25	16.50	159.50	10.00	91.50	28.25	444.87
R5	19.00	24.00	153.50	16.25	105.75	46.50	425.62
R6	45.50	45.75	116.00	30.75	95.00	89.75	395.80
R7	19.75	21.00	174.75	10.75	98.50	29.00	436.79
R8	15.00	17.50	159.25	12.00	113.50	25.50	453.17
R9	13.25	18.75	128.25	9.78	80.50	26.00	471.66
R10	10.75	14.50	117.00	11.92	100.50	19.50	501.99
R11	12.50	15.75	125.25	10.04	96.75	21.00	487.45
R12	14.00	14.25	116.50	9.52	88.50	20.75	483.74
R13	19.00	21.50	126.00	7.84	86.50	26.00	463.14
R14	10.75	15.75	124.75	8.71	95.75	18.50	488.60
R15	14.25	16.75	120.25	8.99	99.25	20.75	482.32
R16	13.25	15.25	123.25	9.52	89.25	19.75	488.88
R17	34.50	33.00	108.50	18.00	83.50	56.50	387.55
P1	157.00	110.25	\	64.50	\	386.25	9.12
P2	171.25	132.50	\	70.75	\	324.75	9.49
P3	193.25	124.25	\	54.25	\	412.25	26.60
P4	191.75	125.25	\	76.50	\	371.50	8.36
P5	155.75	152.75	\	69.25	\	292.75	13.57
P6	182.00	120.25	\	56.25	\	369.00	17.05
P7	193.50	134.25	\	68.25	\	292.50	8.75
P8	205.50	124.75	\	68.25	\	391.50	18.55
P9	172.00	101.00	\	46.36	\	352.50	18.10
P10	176.50	85.75	\	54.35	\	373.25	23.68
P11	176.50	91.75	\	51.61	\	326.00	13.00
P12	186.00	98.75	\	53.76	\	342.50	10.01
P13	170.50	102.25	\	50.78	\	256.00	10.69
P14	194.25	107.50	\	57.48	\	336.25	20.82
P15	182.00	88.75	\	55.37	\	359.75	10.53
P16	179.75	86.75	\	49.00	\	341.00	9.12
P17	177.00	111.75	\	60.25	\	296.00	9.49

Note：R1–R17 and P1–P17 represent the 17 batches of RRR and RRP, respectively; Fru, fructose; Glu, glucose; Suc, sucrose; Mel, melibiose; Raf, raffinose; Mnt, manninotriose; Sta, stachyose; “\”≈0，indicating trace-level content.
